# Supplementary figures and images for: Syntaxin-1A modulates vesicle fusion in mammalian neurons via juxtamembrane domain dependent palmitoylation of its transmembrane domain
Source: eLife. 2022 May 31;11:e78182. doi: 10.7554/eLife.78182 (PMC9183232; doi:10.7554/eLife.78182)

Neuronal lysates  
(Figure 3A)

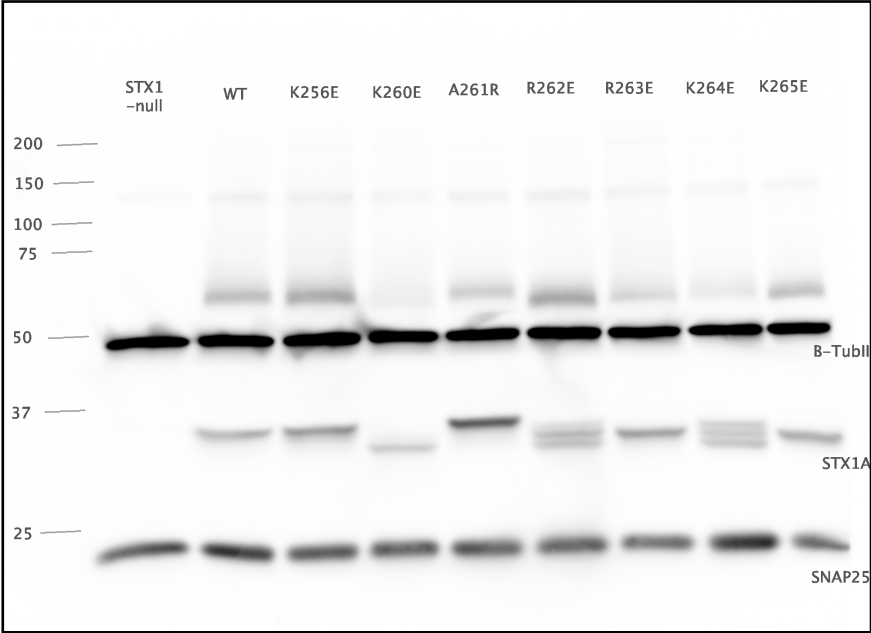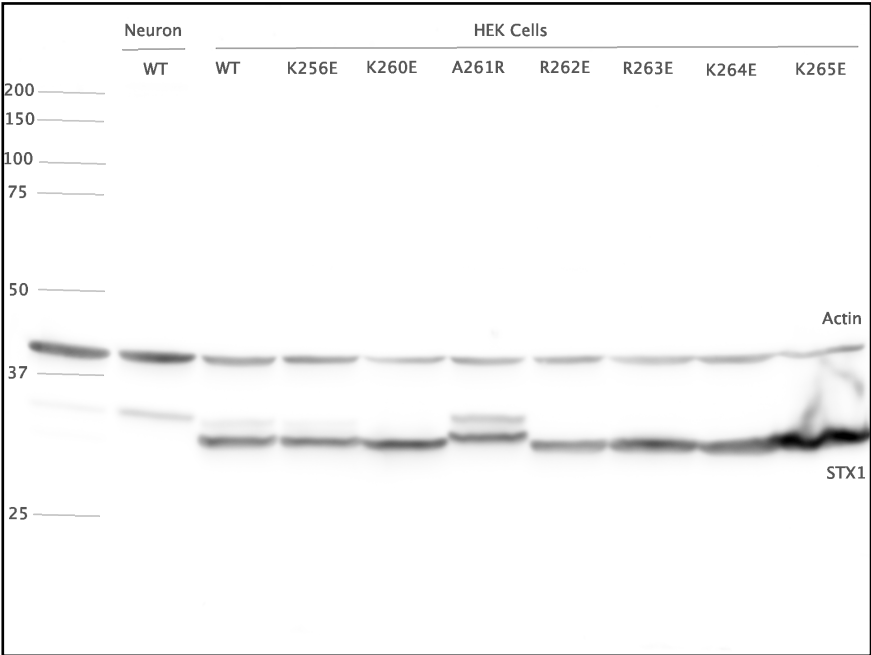

Figure 3 - Source Data 2 -  
whole blots

Supplement: Figure 3—source data 2. [file elife-78182-fig3-data2.zip › Figure 3 - Source Data 2/Figure 3 - Source data 2 - labeled whole blots.pdf]

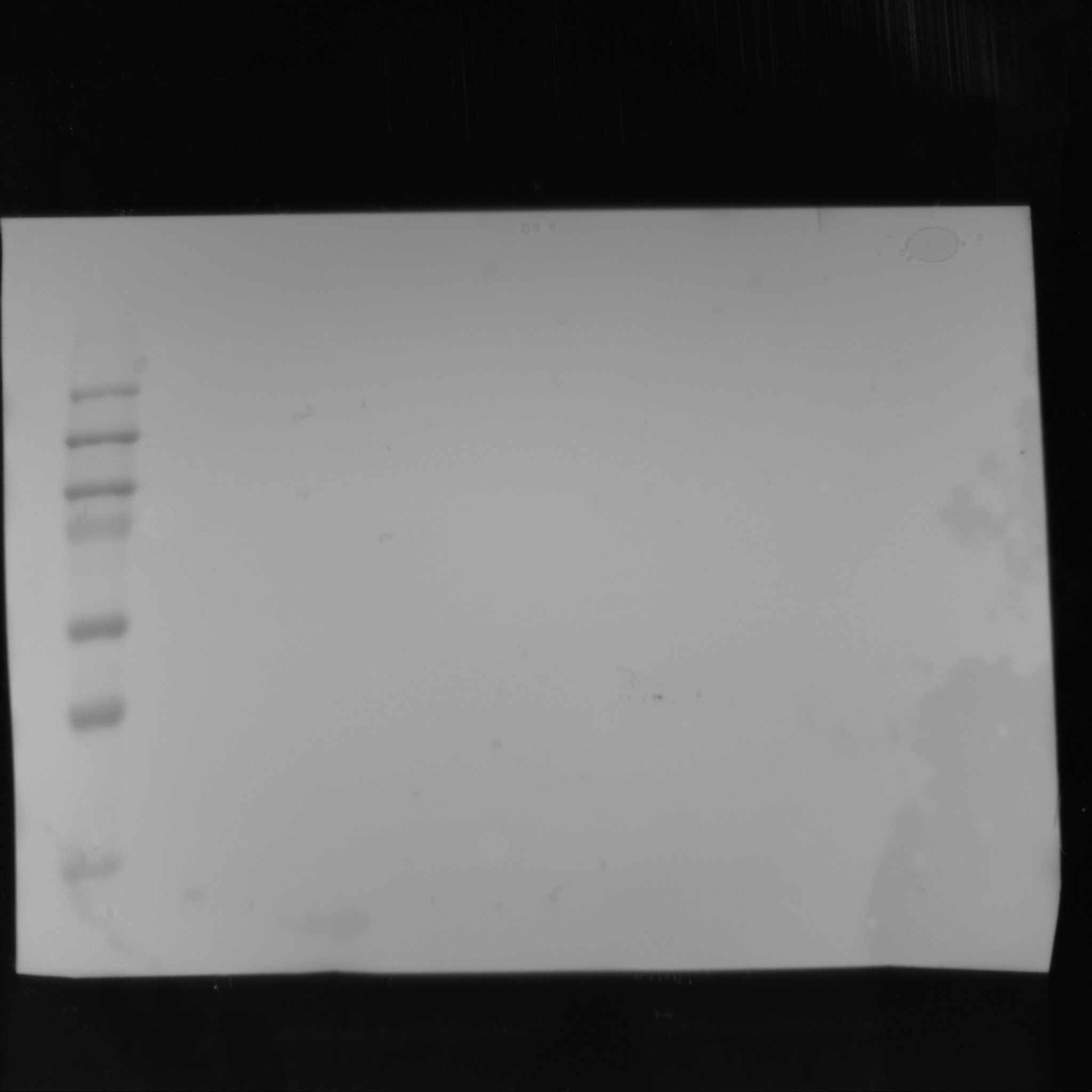

Supplement: Figure 3—source data 2. [file elife-78182-fig3-data2.zip › Figure 3 - Source Data 2/Figure3A_marker_precisionplusKaleidoscope.Tif]

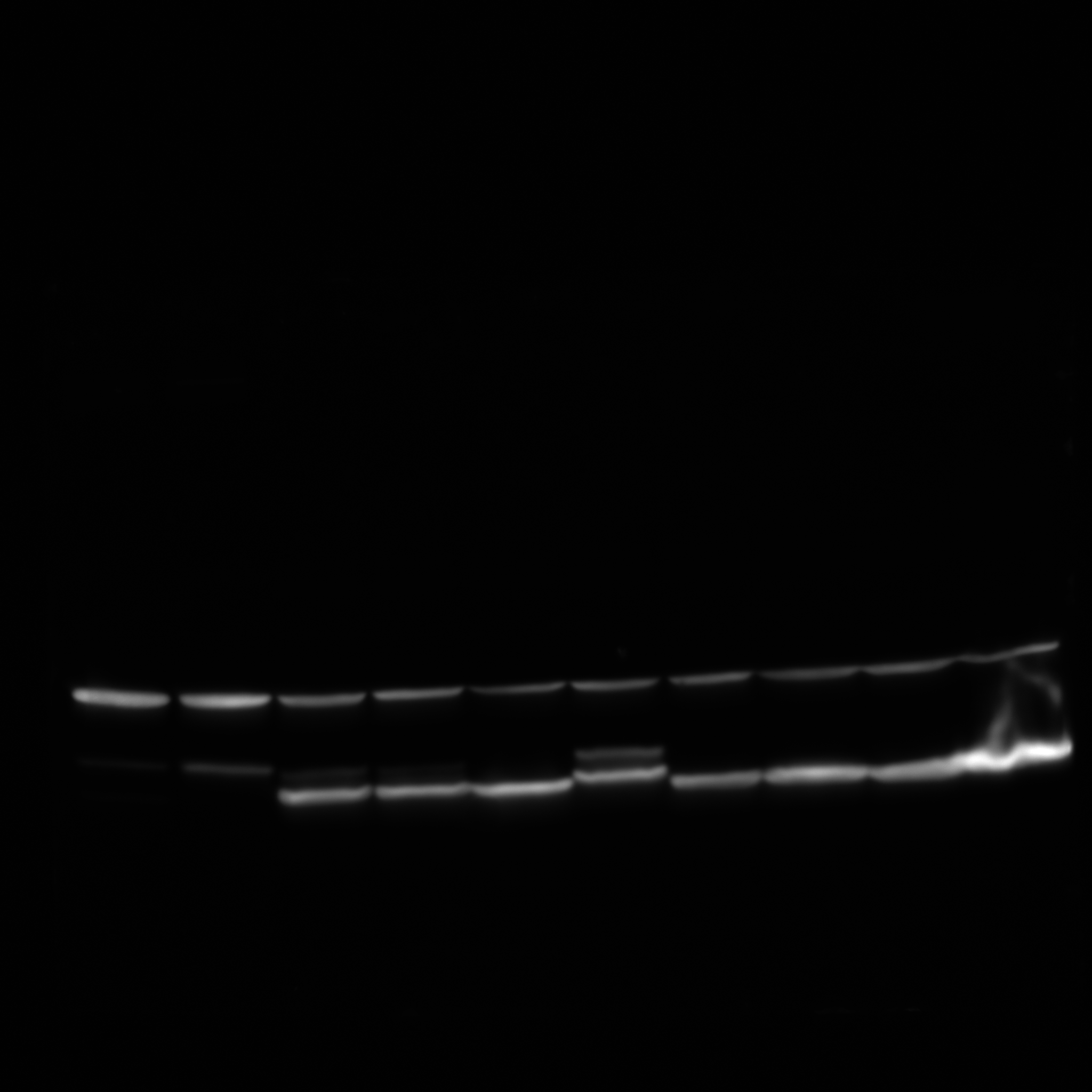

Supplement: Figure 3—source data 2. [file elife-78182-fig3-data2.zip › Figure 3 - Source Data 2/Figure3D_whole_blot.Tif]

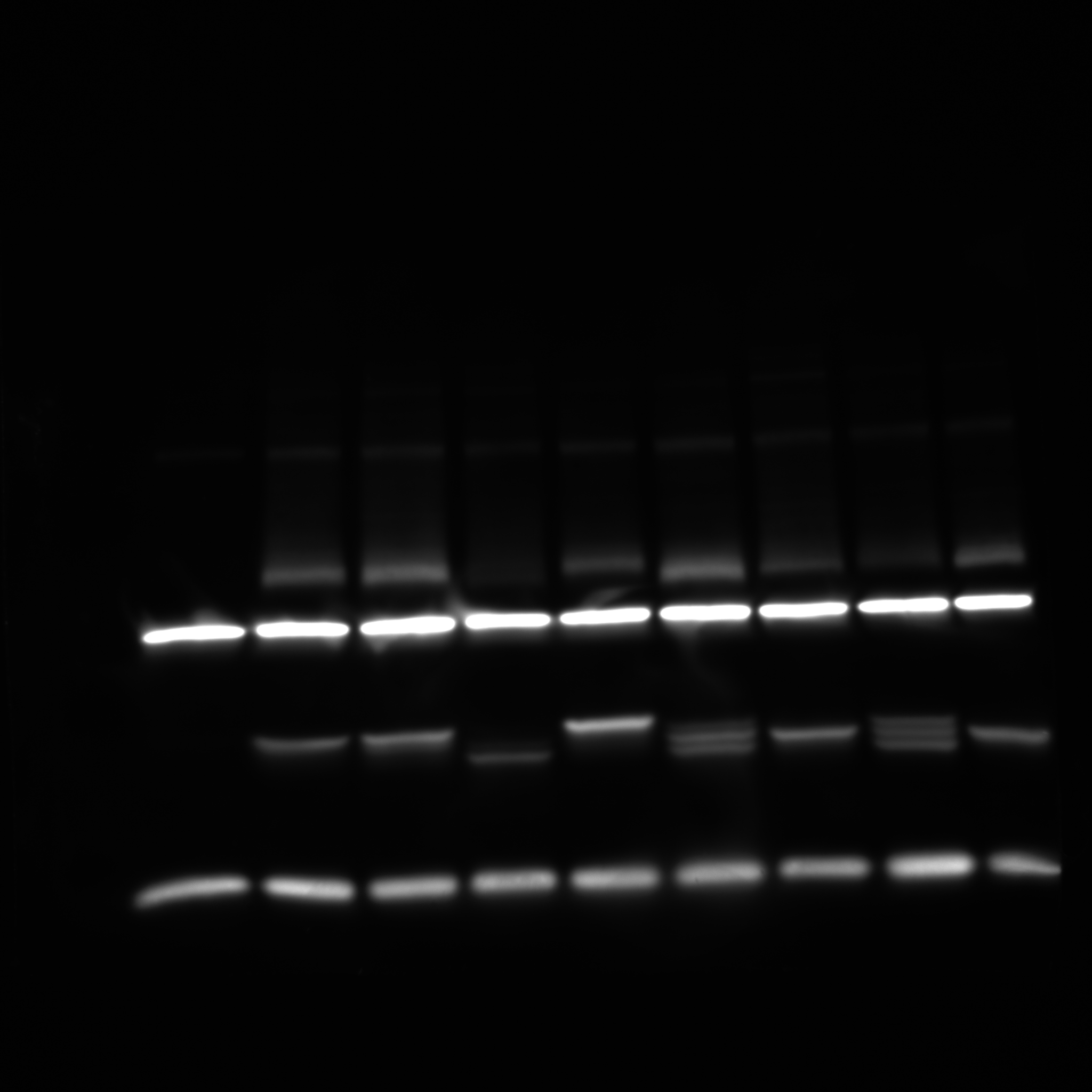

Supplement: Figure 3—source data 2. [file elife-78182-fig3-data2.zip › Figure 3 - Source Data 2/Figure3A_whole_blot.Tif]

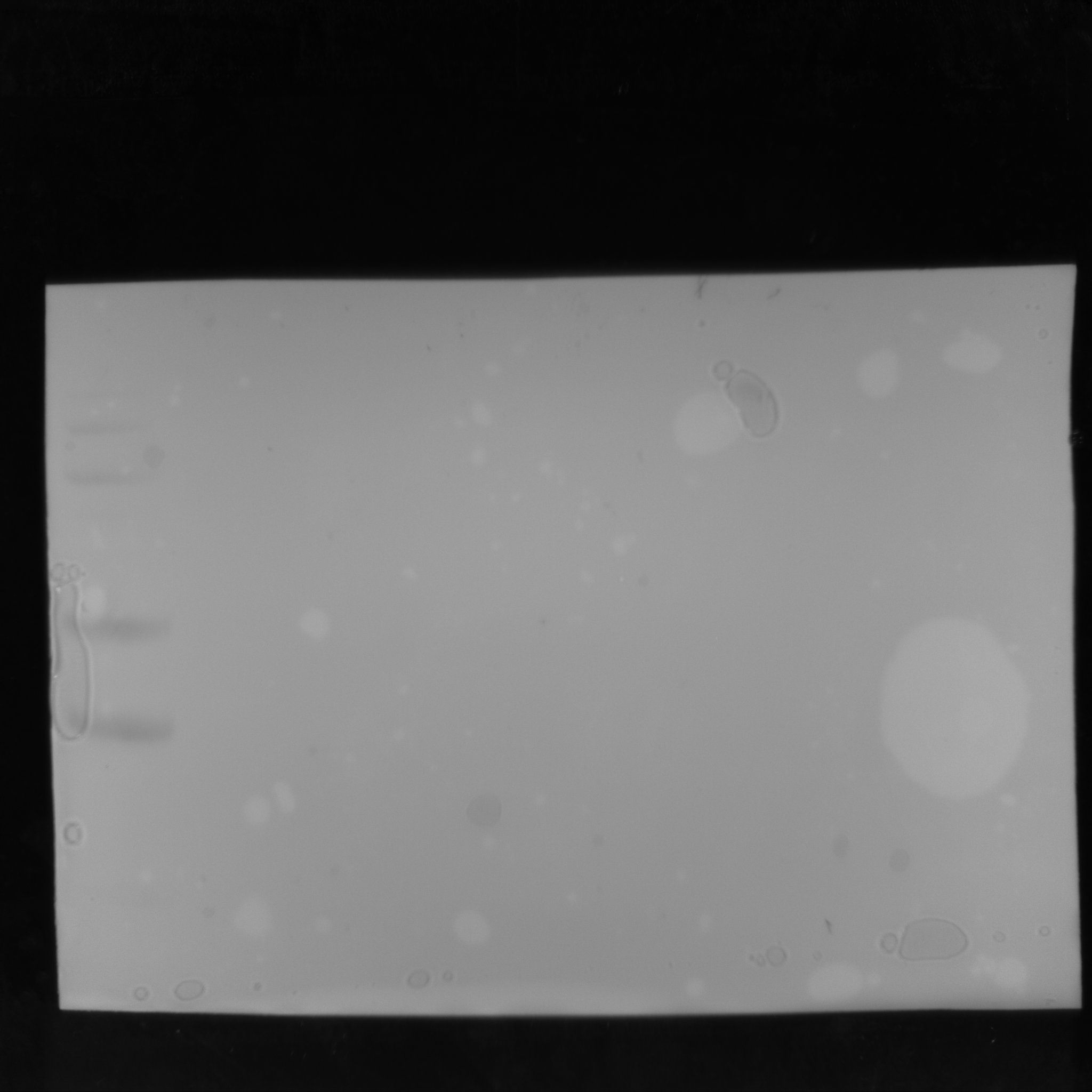

Supplement: Figure 3—source data 2. [file elife-78182-fig3-data2.zip › Figure 3 - Source Data 2/Figure3D_marker_precisionplusKaleidoscope.Tif]

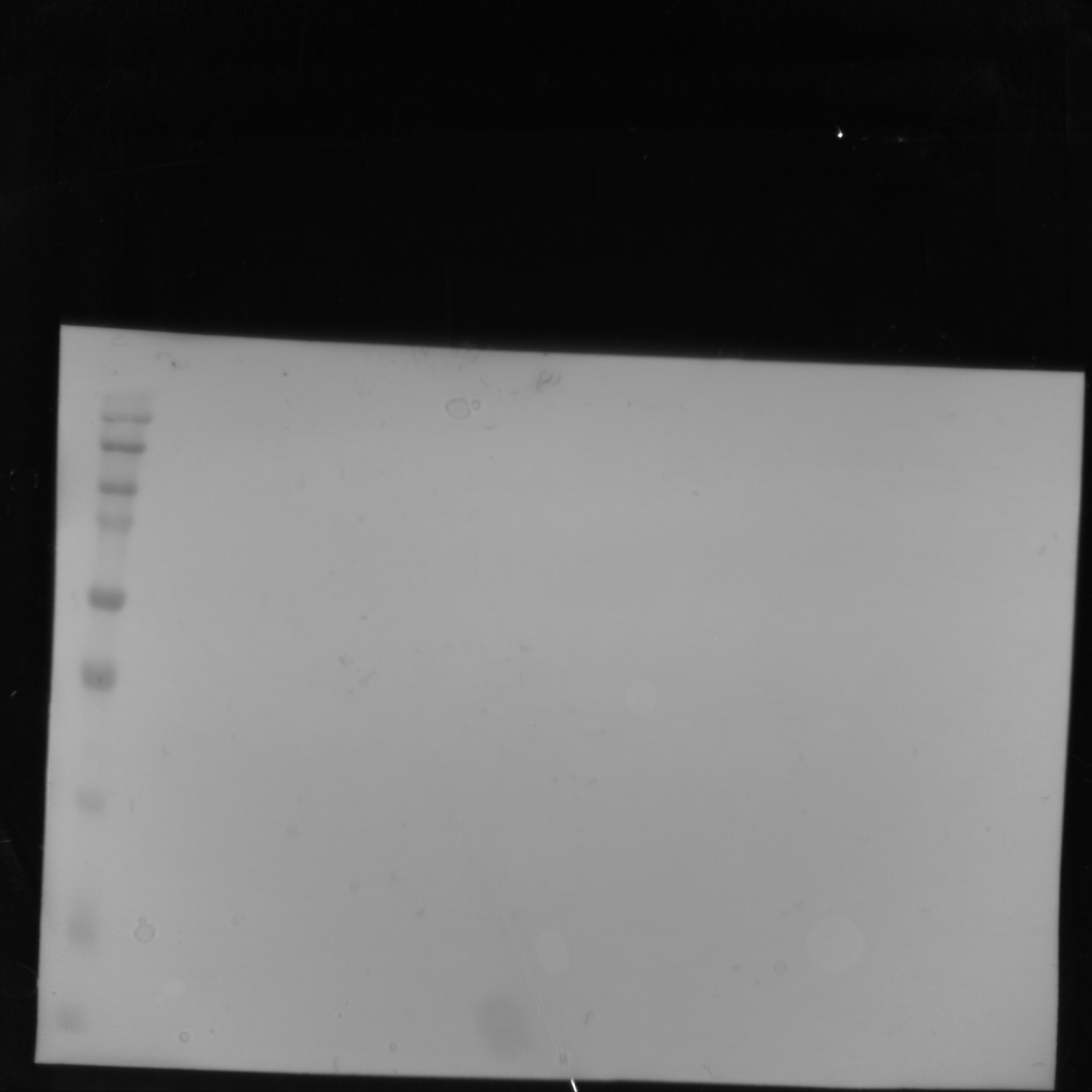

Supplement: Figure 4—source data 2. [file elife-78182-fig4-data2.zip › Figure 4 - Source Data 2/Figure 4D and Figure 4-Supplement 1- Culture 1- whole blot- palmitoylation-precisionplusKaleidoscope.Tif]

**Neuronal lysates**  
**(Figure 4B)**

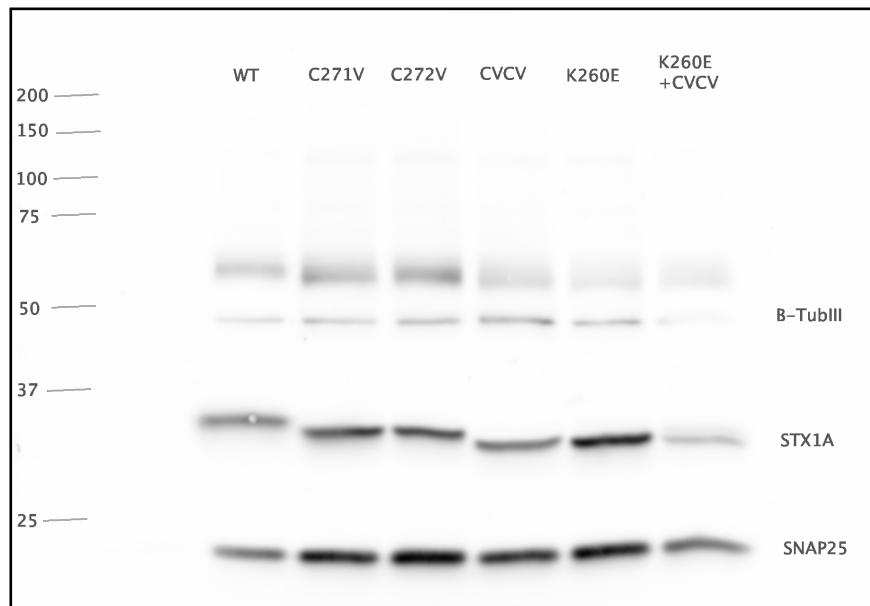

**Figure 4 - Source Data 2 -  
whole blot**

Supplement: Figure 4—source data 2. [file elife-78182-fig4-data2.zip › Figure 4 - Source Data 2/Figure 4B labeled whole blot.pdf]

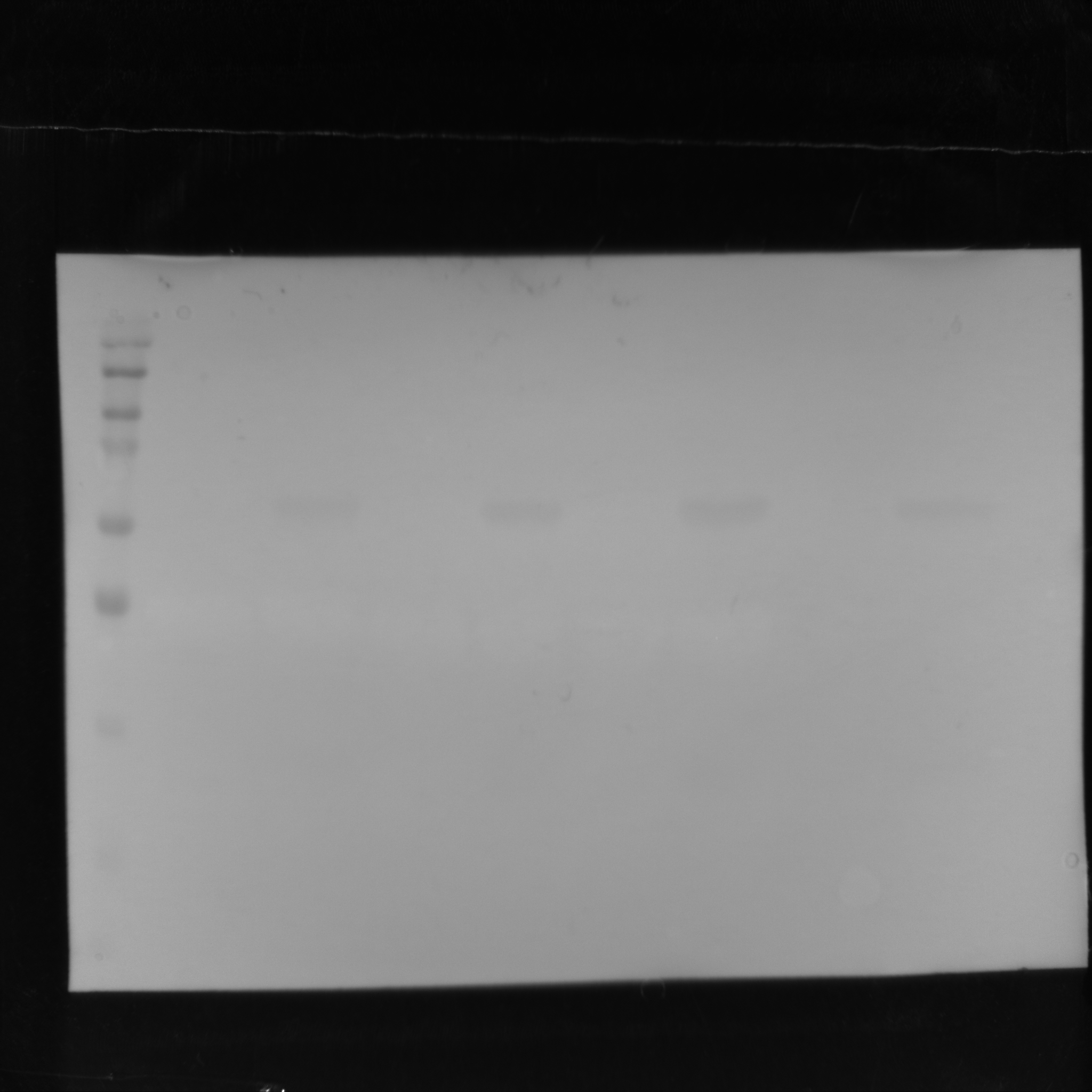

Supplement: Figure 4—source data 2. [file elife-78182-fig4-data2.zip › Figure 4 - Source Data 2/Figure 4D and Figure 4-Supplement 1- Culture 1- whole blot- reprobeSTX1A-precisionplusKaleidoscope.Tif]

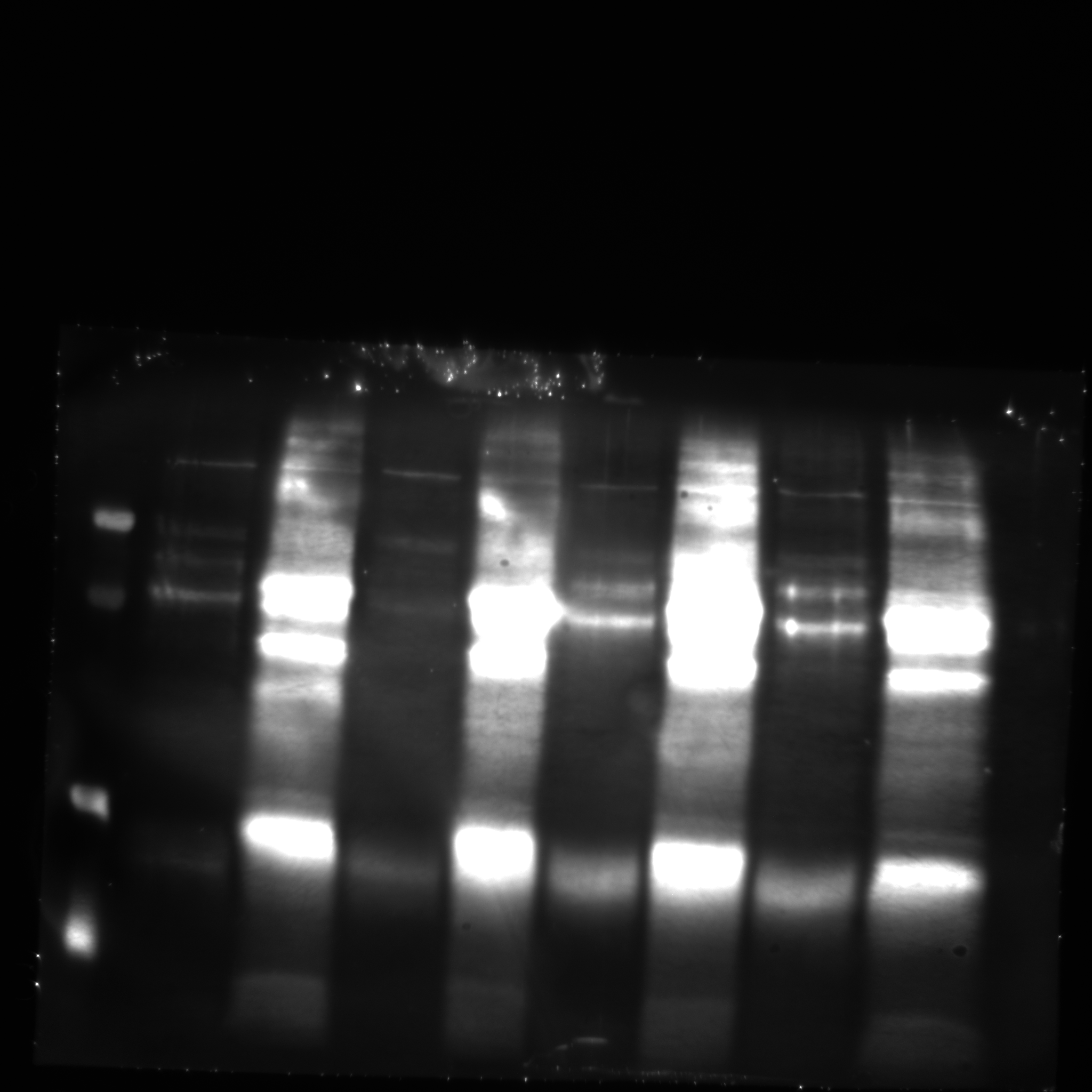

Supplement: Figure 4—source data 2. [file elife-78182-fig4-data2.zip › Figure 4 - Source Data 2/Figure 4D and Figure 4-Supplement 1- Culture 1- whole blot- palmitoylation.Tif]

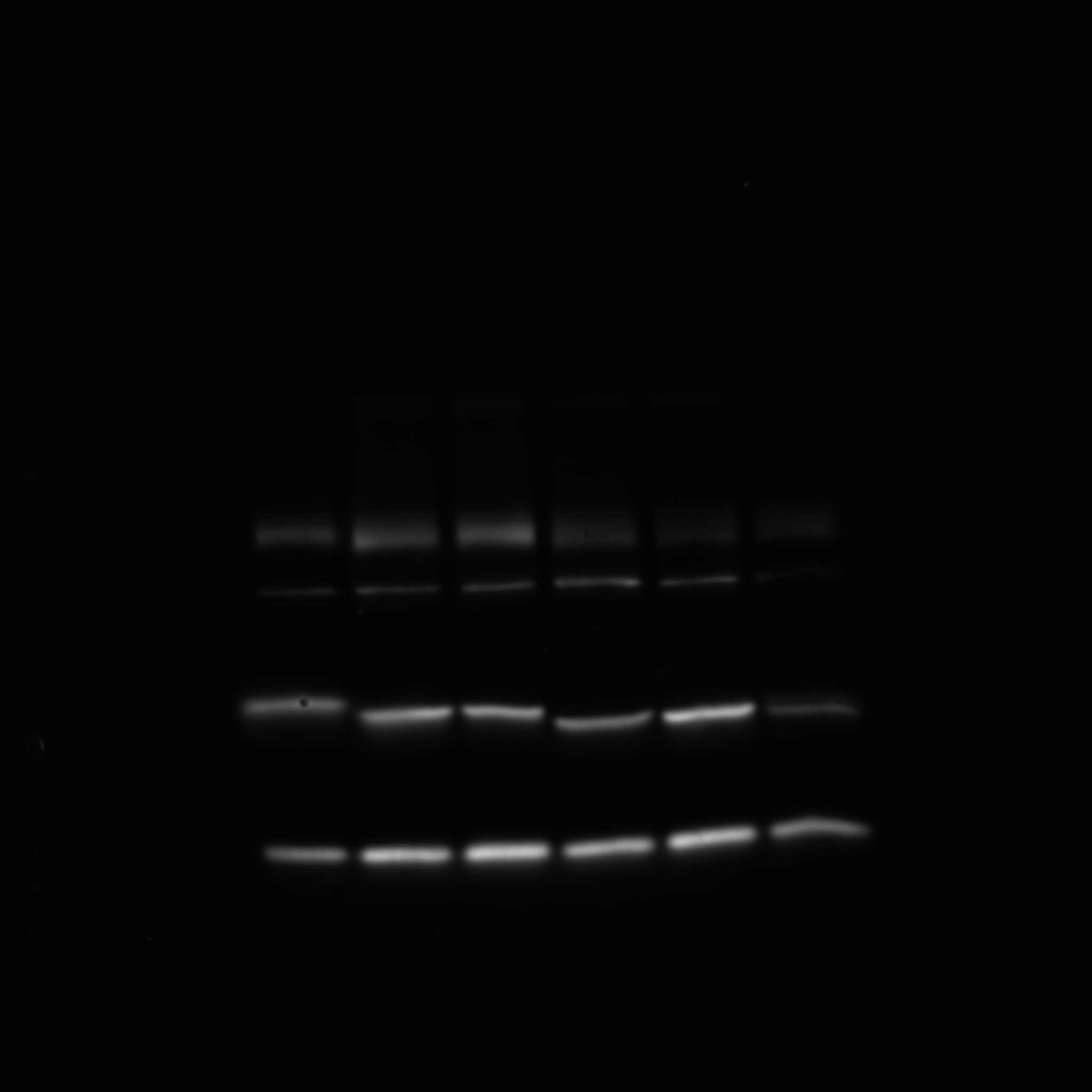

Supplement: Figure 4—source data 2. [file elife-78182-fig4-data2.zip › Figure 4 - Source Data 2/Figure4B_whole_blot.Tif]

Culture 1

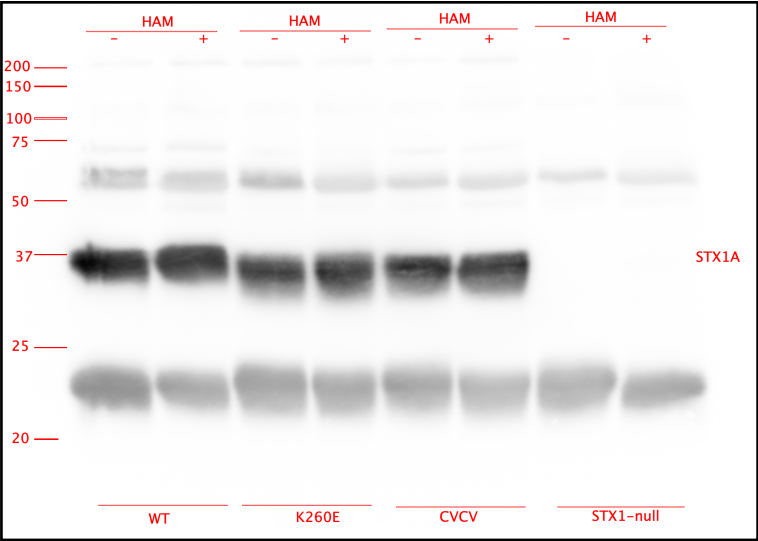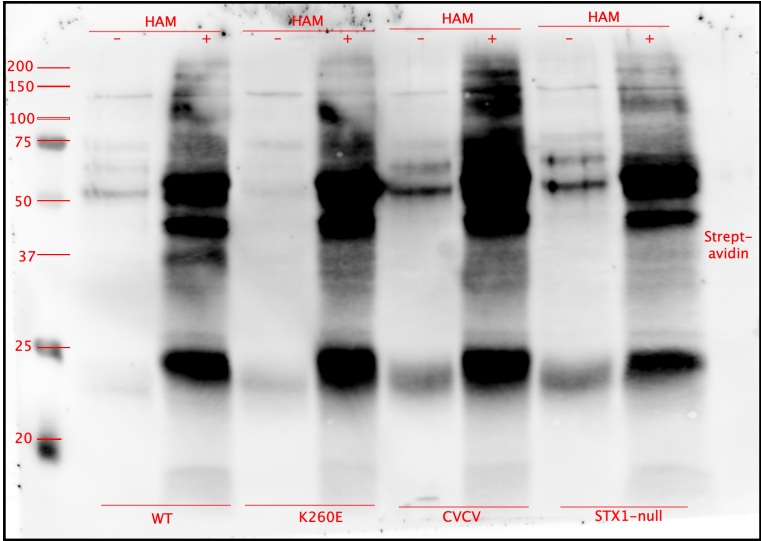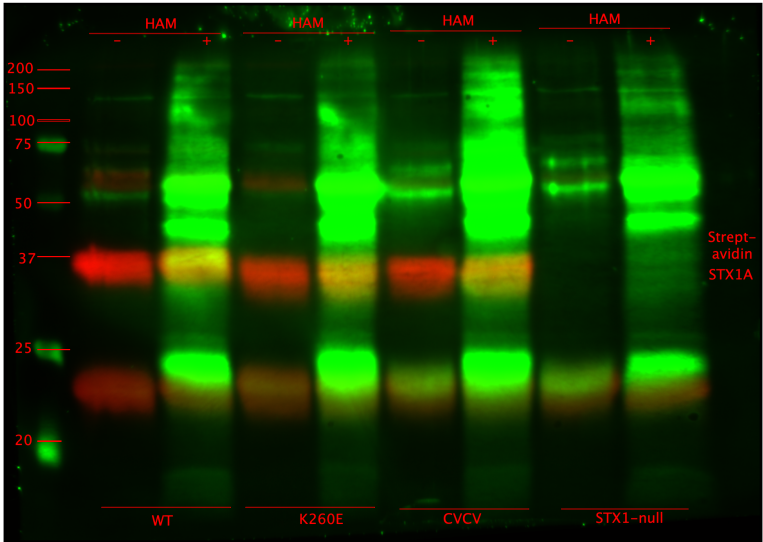

Supplement: Figure 4—source data 2. [file elife-78182-fig4-data2.zip › Figure 4 - Source Data 2/Figure 4D and Figure 4-Supplement 1- Culture 1- labeled whole blots.pdf]

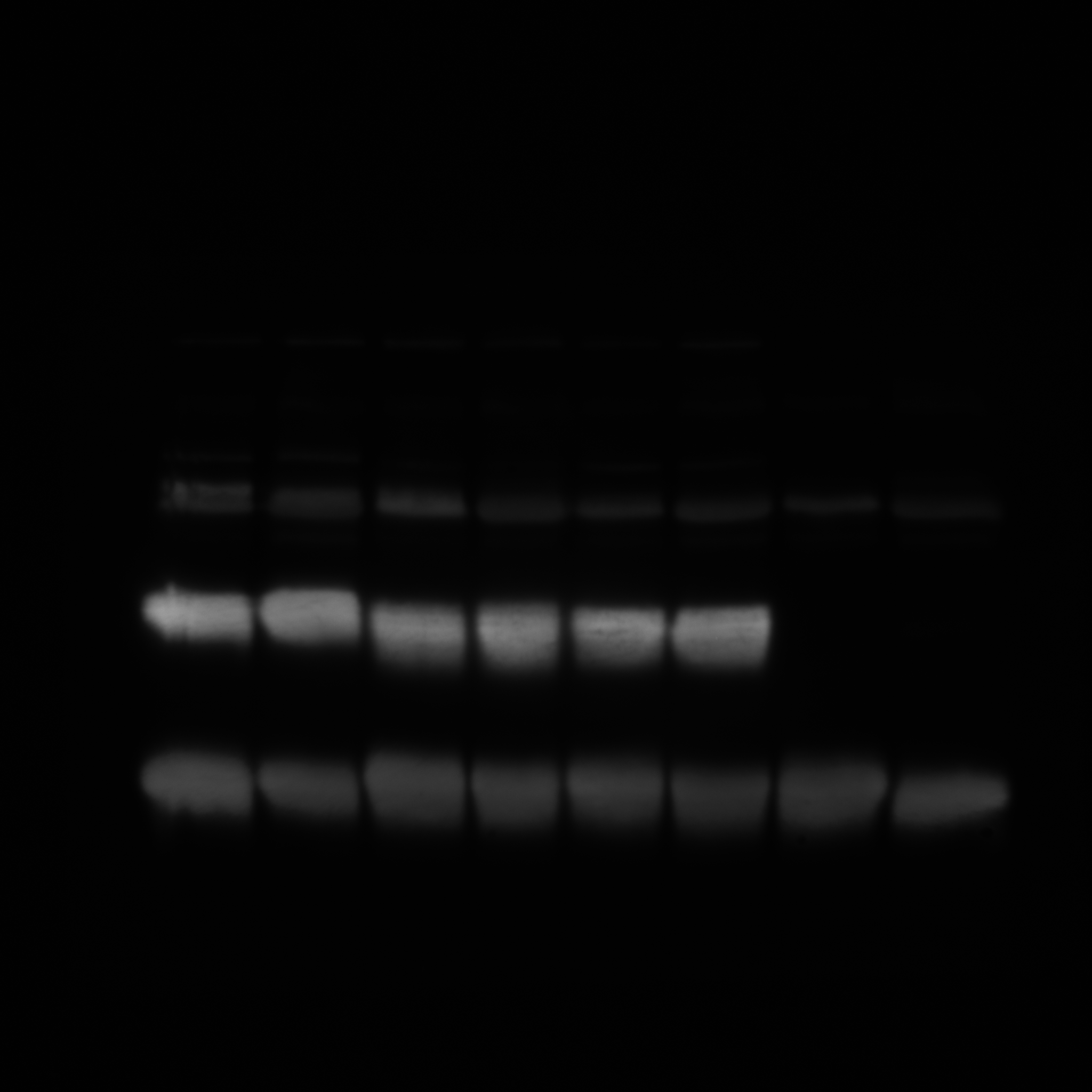

Supplement: Figure 4—source data 2. [file elife-78182-fig4-data2.zip › Figure 4 - Source Data 2/Figure 4D and Figure 4-Supplement 1- Culture 1- whole blot- reprobeSTX1A.Tif]

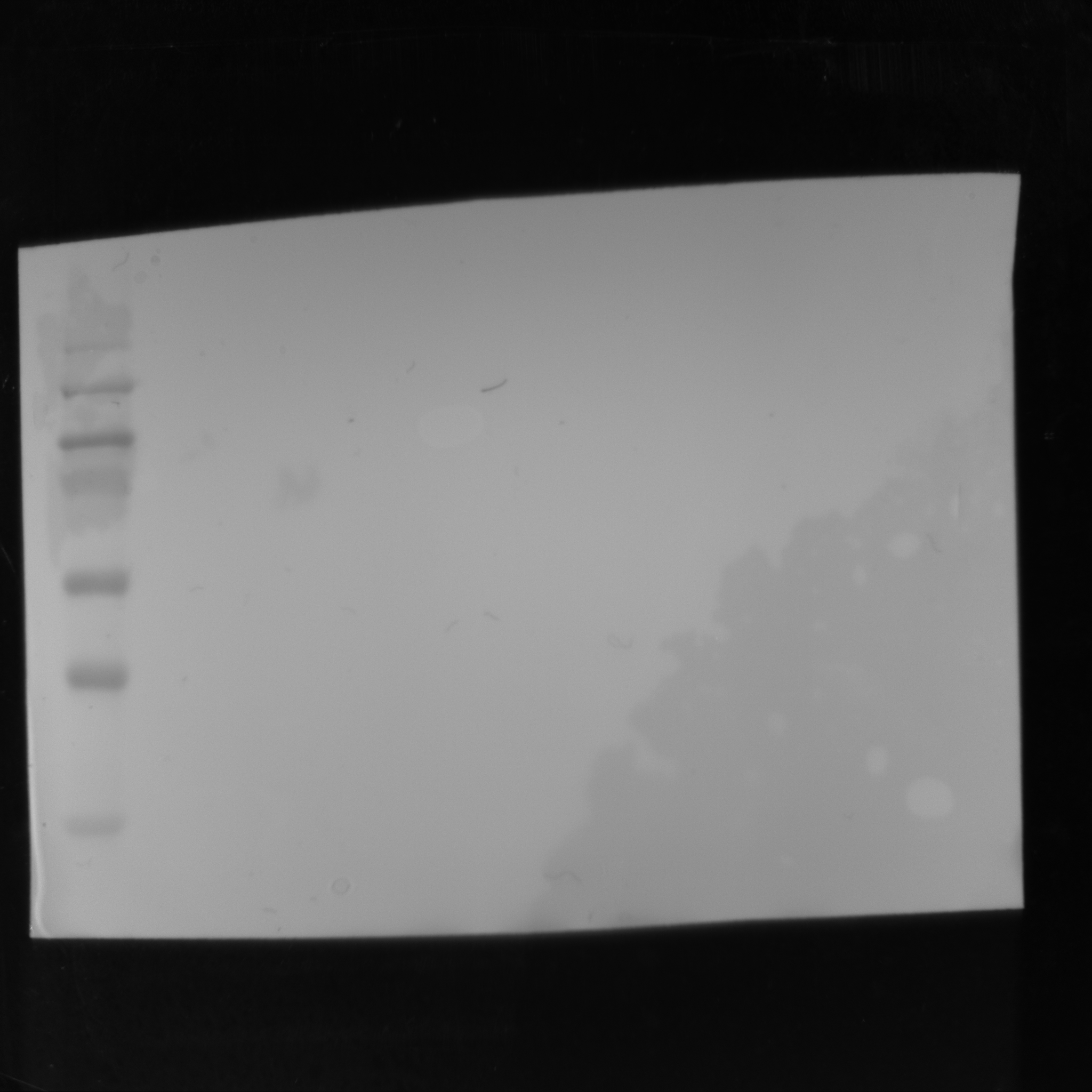

Supplement: Figure 4—source data 2. [file elife-78182-fig4-data2.zip › Figure 4 - Source Data 2/Figure4B_marker_precisionplusKaleidoscope.Tif.Tif]

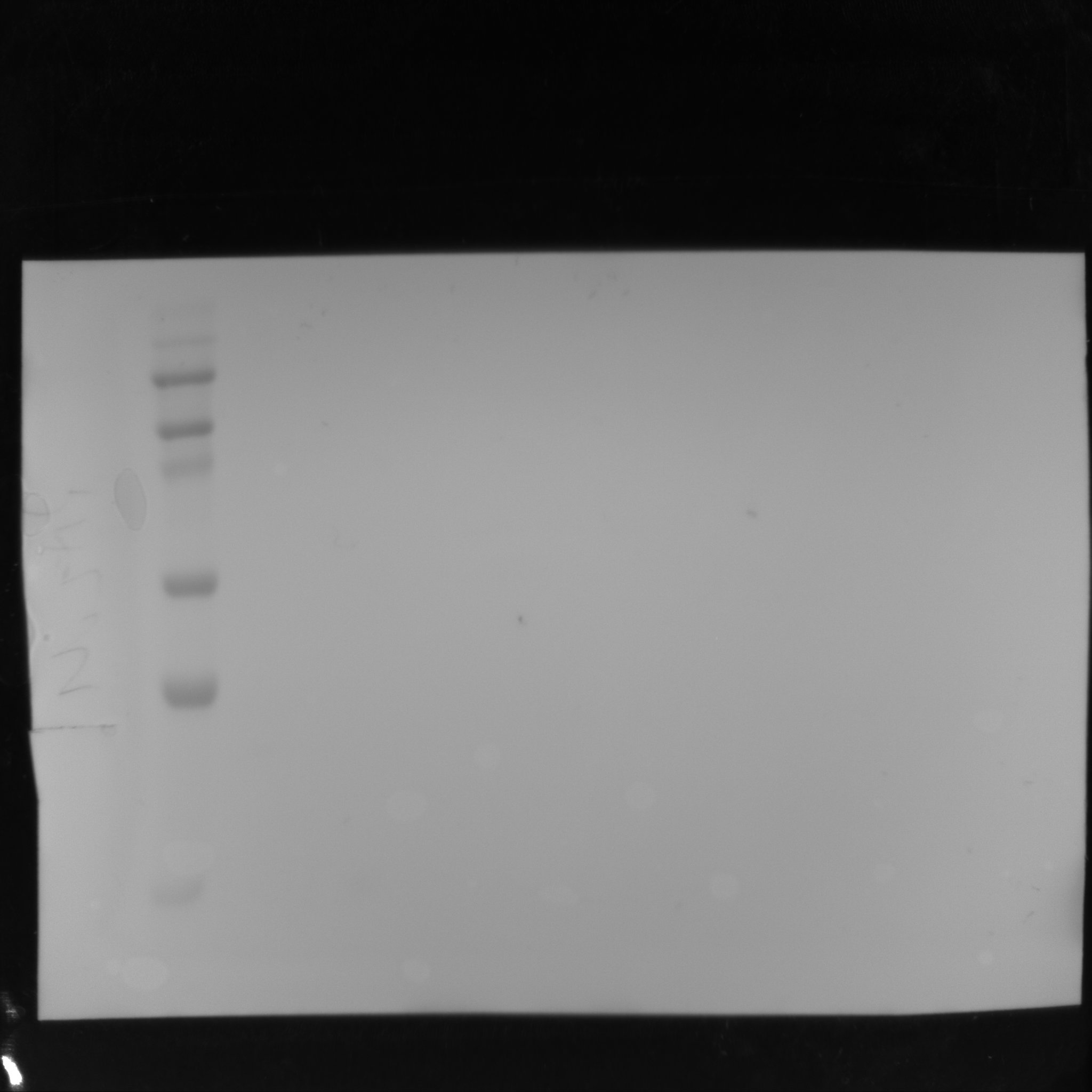

Supplement: Figure 4—figure supplement 1—source data 1. [file elife-78182-fig4-figsupp1-data1.zip › Figure 4 - Supplement 1 -Source Data 1/Figure 4-Supplement 1-Culture2- whole blot- palmitoylation-precisionplusKaleidoscope.Tif]

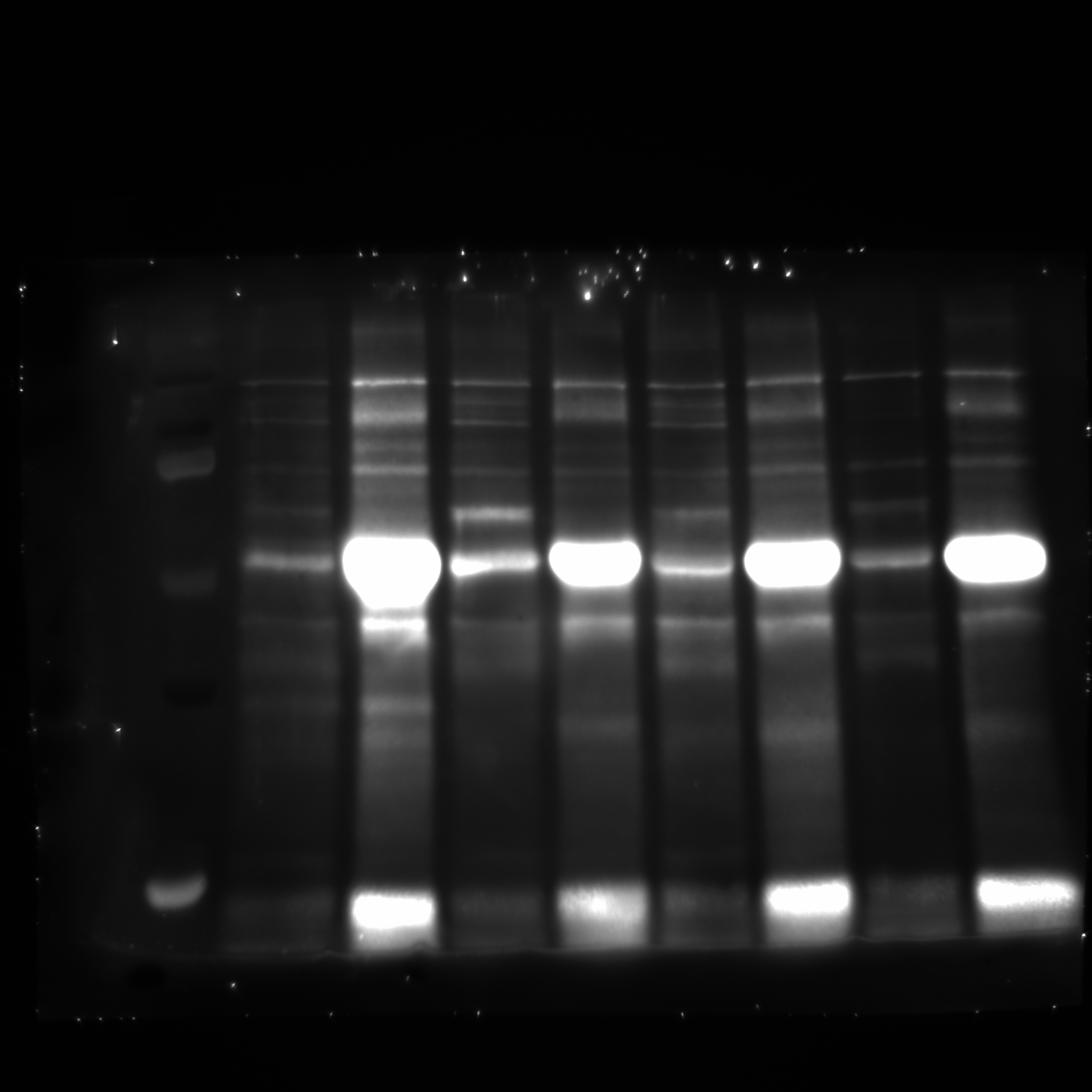

Supplement: Figure 4—figure supplement 1—source data 1. [file elife-78182-fig4-figsupp1-data1.zip › Figure 4 - Supplement 1 -Source Data 1/Figure 4-Supplement 1-Culture2- whole blot- palmitoylation.Tif]

Culture 2

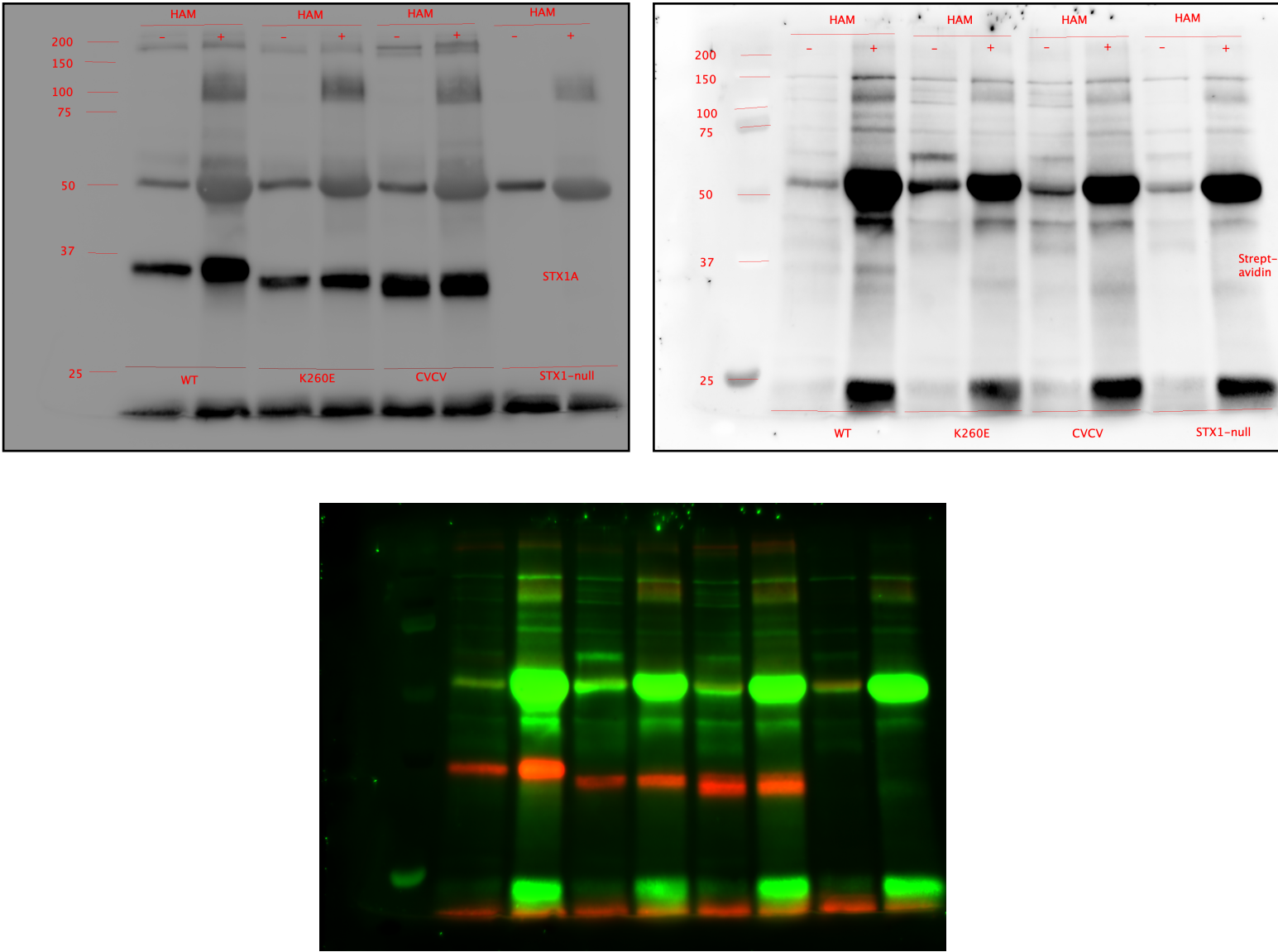

Figure 4 - Supplement 1 -  
Source Data 1 - whole blots

Supplement: Figure 4—figure supplement 1—source data 1. [file elife-78182-fig4-figsupp1-data1.zip › Figure 4 - Supplement 1 -Source Data 1/Figure 4-Supplement 1 - labeled whole blots.pdf.pdf]

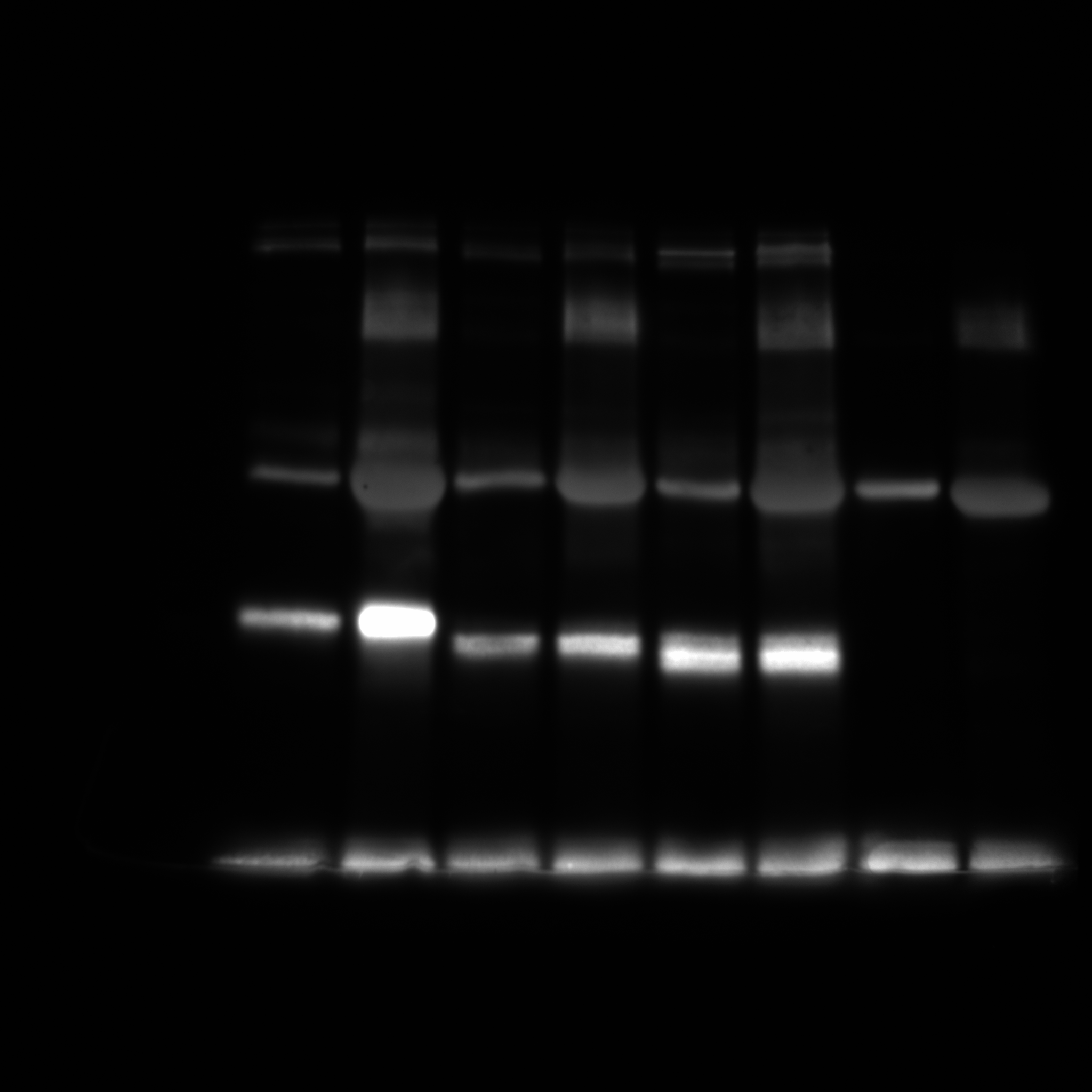

Supplement: Figure 4—figure supplement 1—source data 1. [file elife-78182-fig4-figsupp1-data1.zip › Figure 4 - Supplement 1 -Source Data 1/Figure 4-Supplement 1-Culture2- whole blot- reprobeSTX1A.Tif]

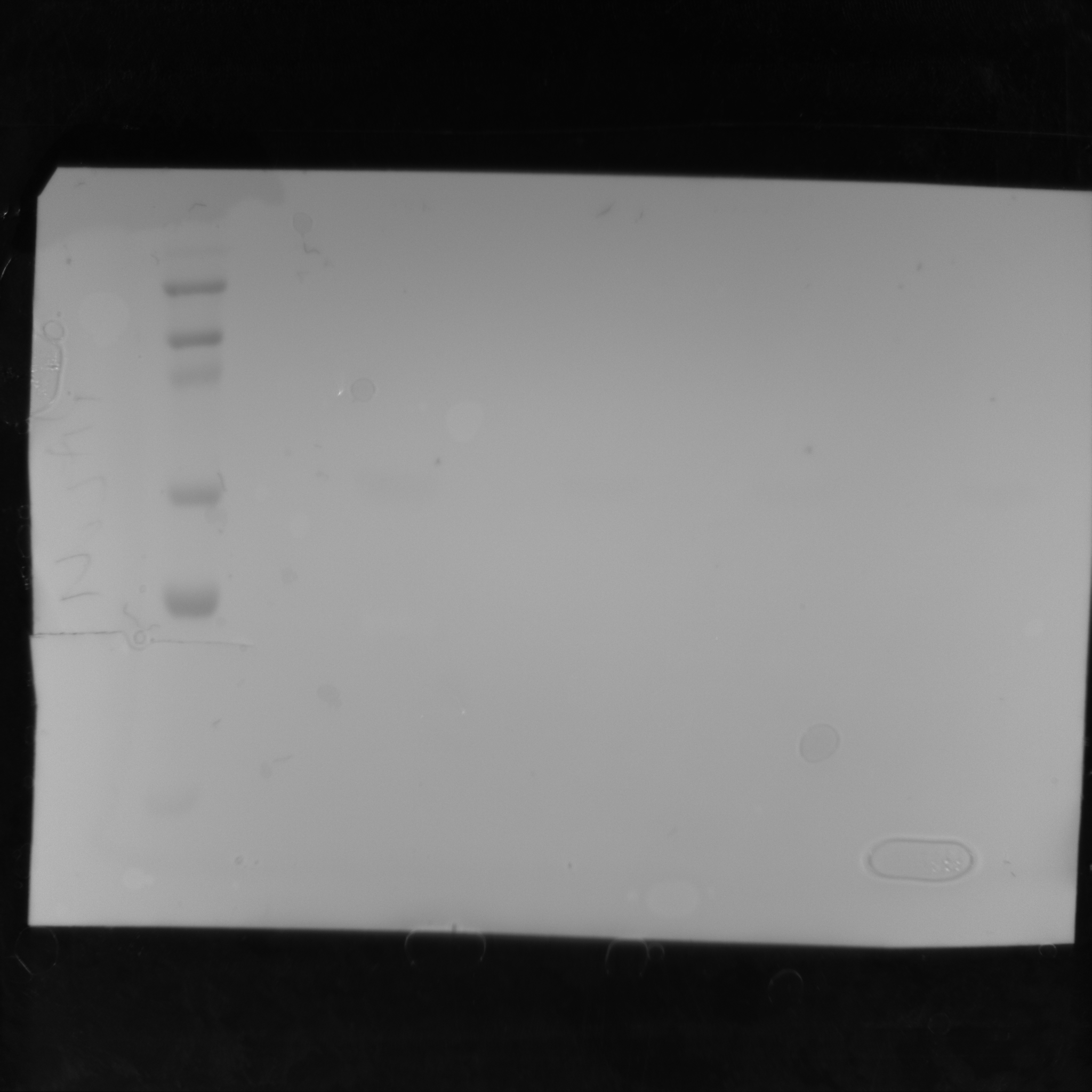

Supplement: Figure 4—figure supplement 1—source data 1. [file elife-78182-fig4-figsupp1-data1.zip › Figure 4 - Supplement 1 -Source Data 1/Figure 4-Supplement 1-Culture2- whole blot- reprobeSTX1A-precisionplusKaleidoscope.Tif.Tif]

Neuronal lysates  
(Figure 6B)

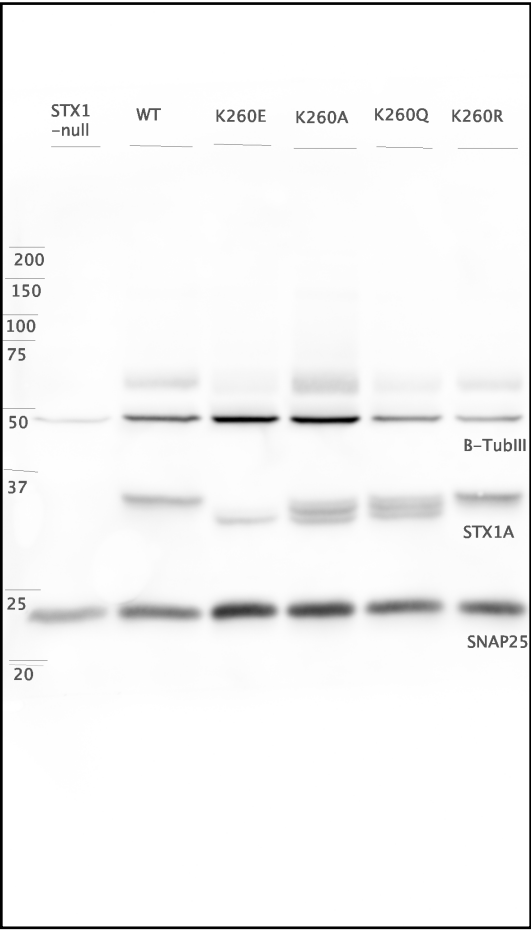

Figure 6 - Source Data 2 - whole blot

Supplement: Figure 6—source data 2. [file elife-78182-fig6-data2.zip › Figure 6 - Source Data 2/Figure6B Source data 2 labeled whole blot.pdf]

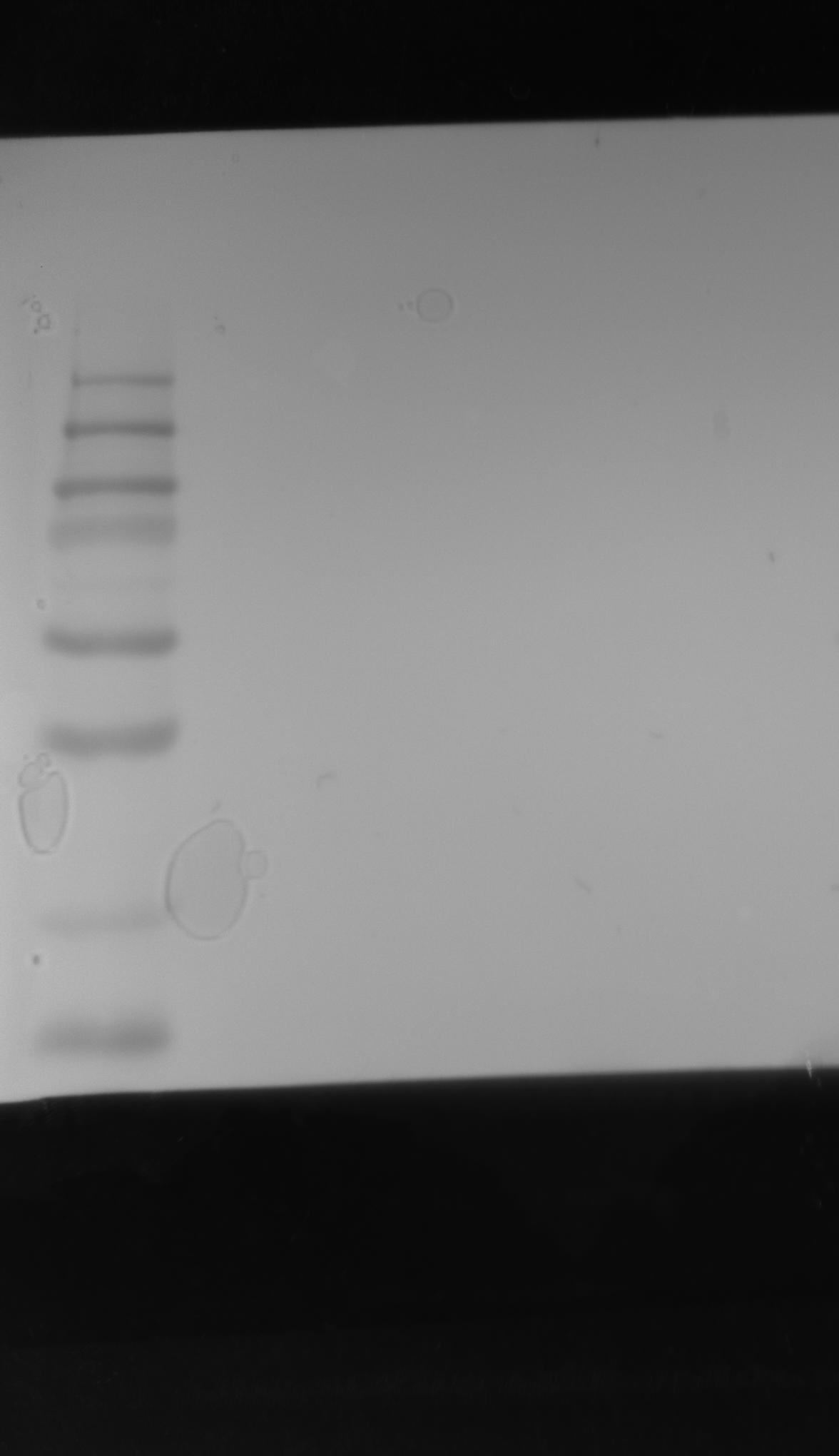

Supplement: Figure 6—source data 2. [file elife-78182-fig6-data2.zip › Figure 6 - Source Data 2/Figure6B_marker_precisionplusKaleidoscope.tif]

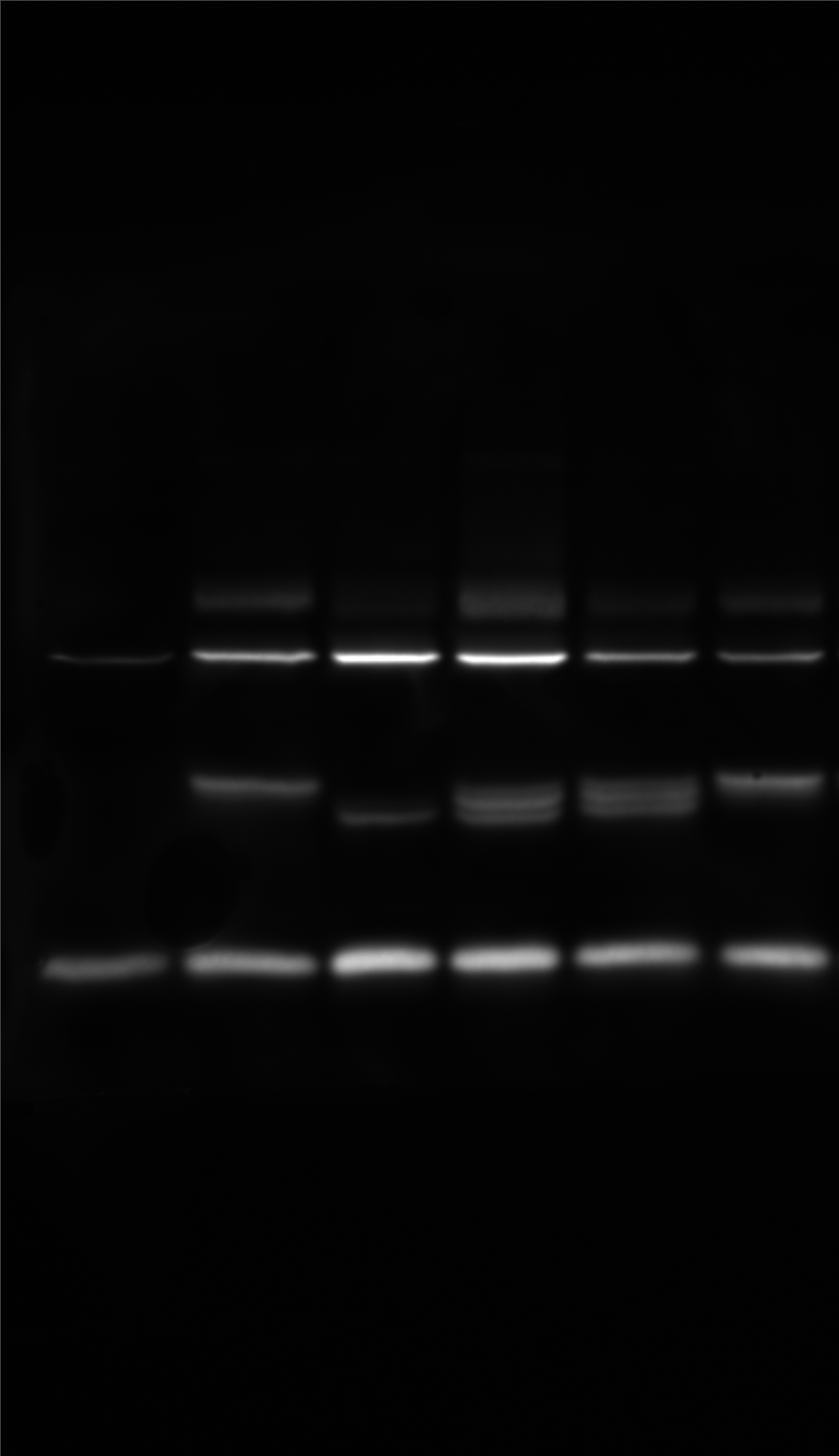

Supplement: Figure 6—source data 2. [file elife-78182-fig6-data2.zip › Figure 6 - Source Data 2/Figure6B_whole_blot.tif]

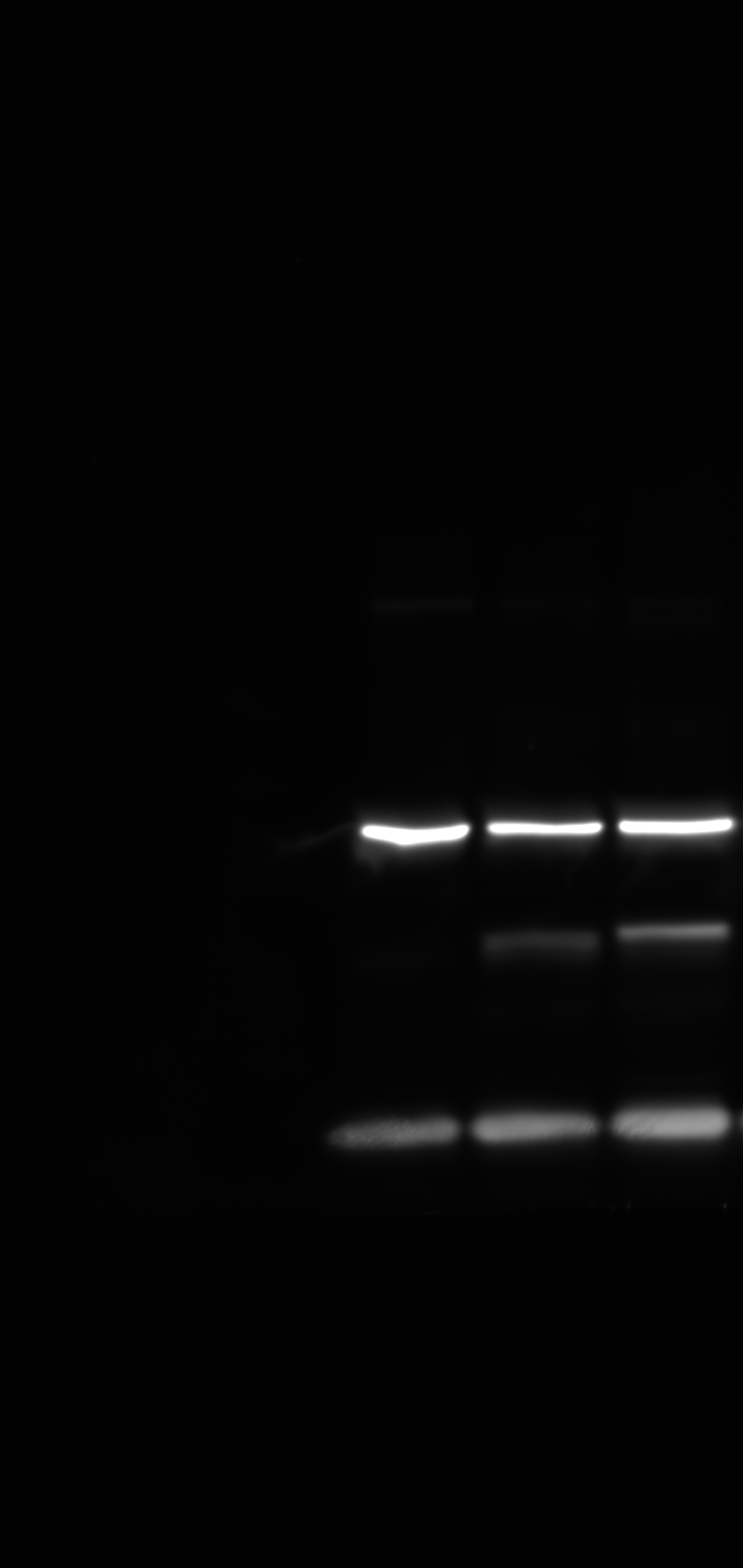

Supplement: Figure 7—source data 2. [file elife-78182-fig7-data2.zip › Figure 7 - Source Data 2/Figure7B_whole_blot.tif]

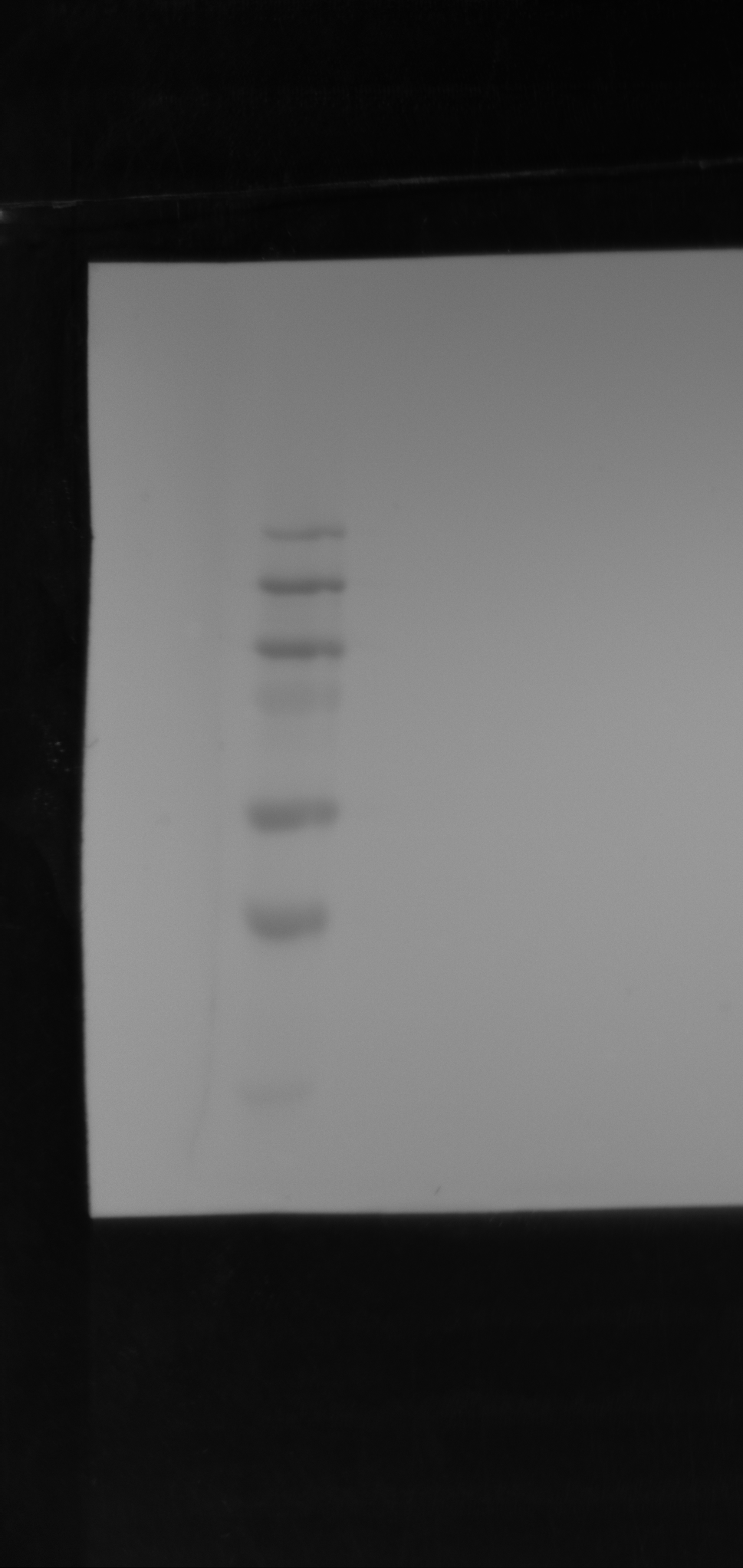

Supplement: Figure 7—source data 2. [file elife-78182-fig7-data2.zip › Figure 7 - Source Data 2/Figure7B_marker_precisionplusKaleidoscope.Tif]

Neuronal lysates  
(Figure 7B)

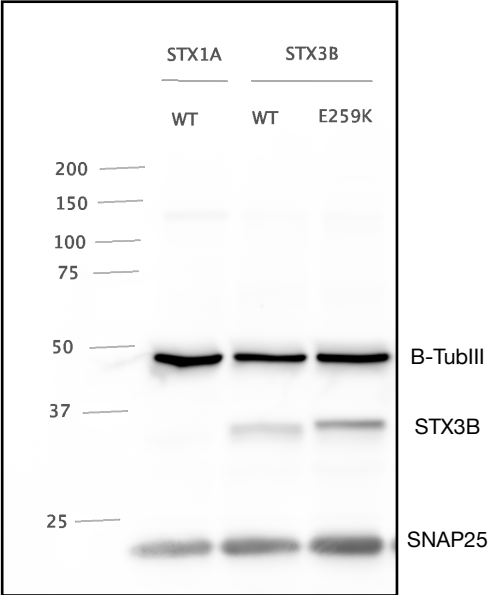

Figure 7 - Source Data 2 - whole blot

Supplement: Figure 7—source data 2. [file elife-78182-fig7-data2.zip › Figure 7 - Source Data 2/Figure7B labeled whole blot.pdf]

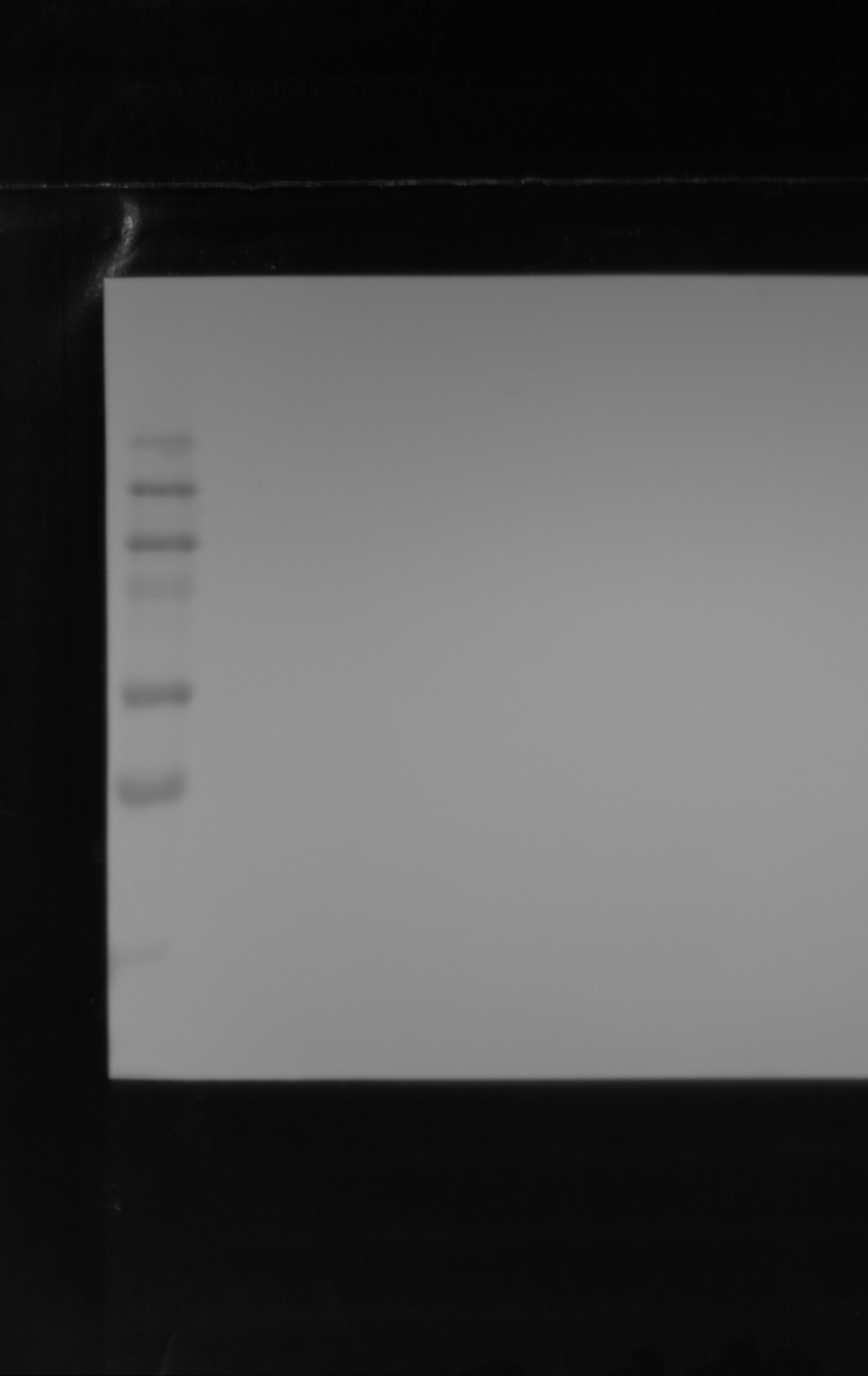

Supplement: Figure 8—source data 2. [file elife-78182-fig8-data2.zip › Figure 8 - Source Data 2/Figure8B_marker_precisionplusKaleidoscope.tif]

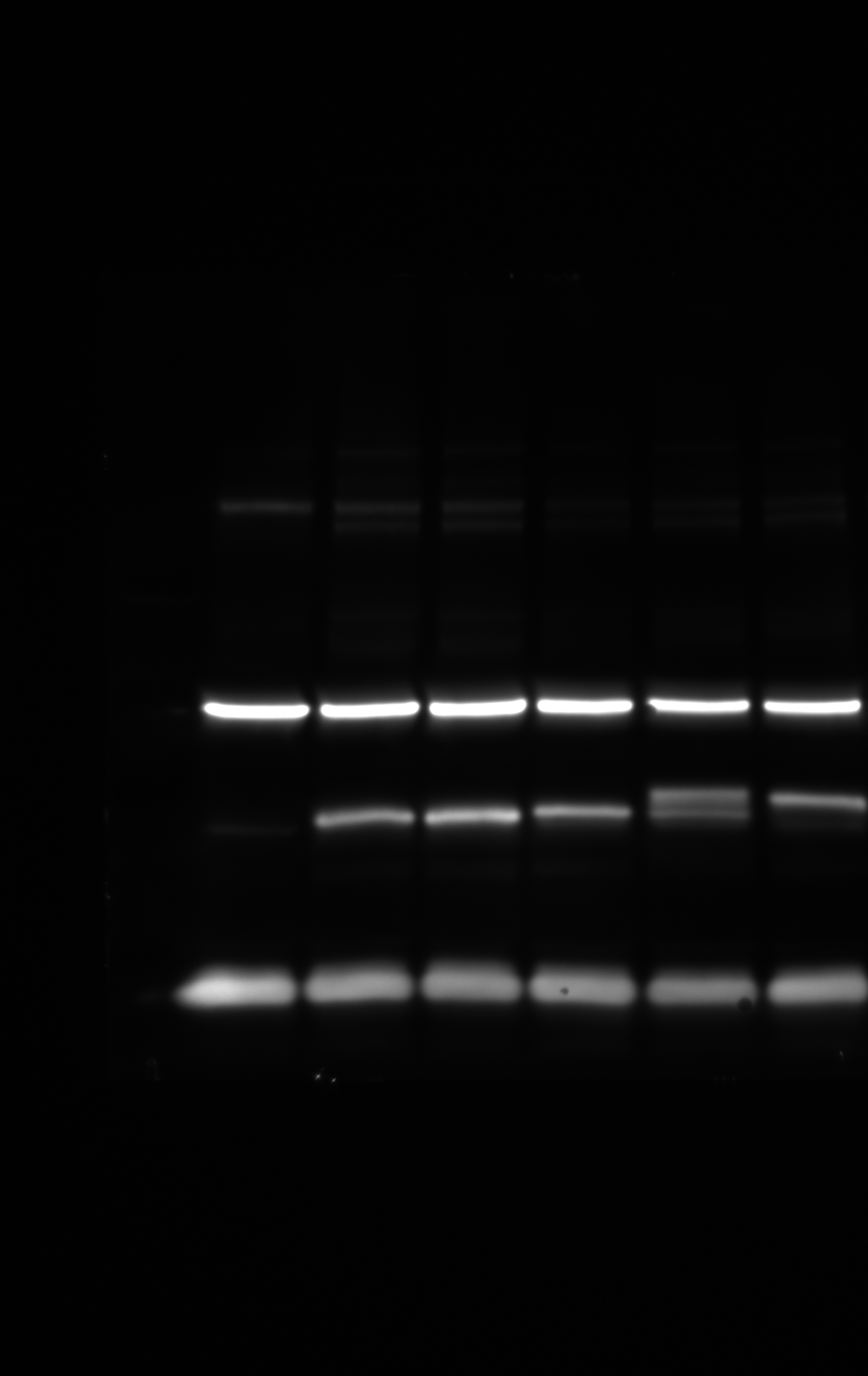

Supplement: Figure 8—source data 2. [file elife-78182-fig8-data2.zip › Figure 8 - Source Data 2/Figure8B_whole_blot.tif]

Neuronal lysates  
(Figure 8B)

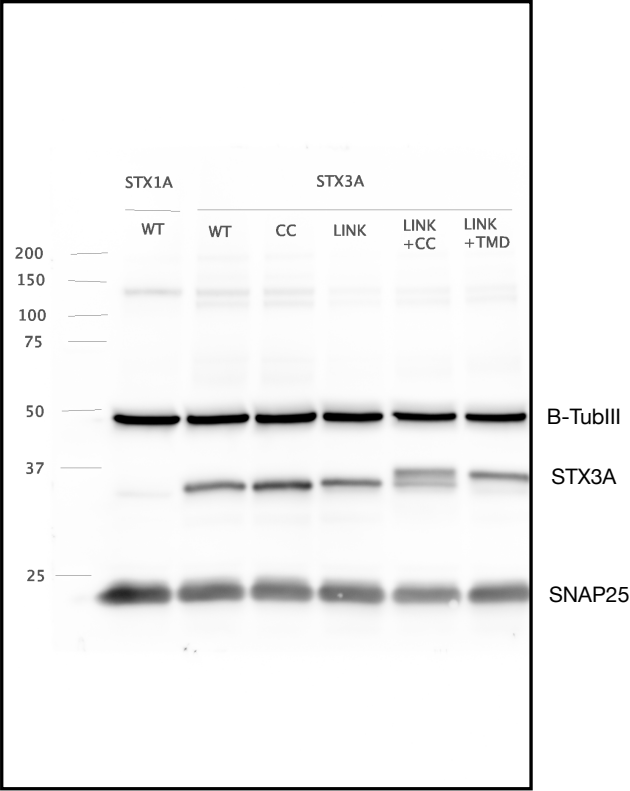

Figure 8 - Source Data 2 -  
whole blot

Supplement: Figure 8—source data 2. [file elife-78182-fig8-data2.zip › Figure 8 - Source Data 2/Figure 8B - Source data 2 - labeled whole blot.pdf]
